# Epigenetic Alterations Are Critical for Fear Memory Consolidation and Synaptic Plasticity in the Lateral Amygdala

**DOI:** 10.1371/journal.pone.0019958

**Published:** 2011-05-20

**Authors:** Melissa S. Monsey, Kristie T. Ota, Irene F. Akingbade, Ellie S. Hong, Glenn E. Schafe

**Affiliations:** 1 Department of Psychology, Yale University, New Haven, Connecticut, United States of America; 2 Interdepartmental Neuroscience Program, Yale University, New Haven, Connecticut, United States of America; Pontifical Catholic University of Rio Grande, Brazil

## Abstract

Epigenetic mechanisms, including histone acetylation and DNA methylation, have been widely implicated in hippocampal-dependent learning paradigms. Here, we have examined the role of epigenetic alterations in amygdala-dependent auditory Pavlovian fear conditioning and associated synaptic plasticity in the lateral nucleus of the amygdala (LA) in the rat. Using Western blotting, we first show that auditory fear conditioning is associated with an increase in histone H3 acetylation and DNMT3A expression in the LA, and that training-related alterations in histone acetylation and DNMT3A expression in the LA are downstream of ERK/MAPK signaling. Next, we show that intra-LA infusion of the histone deacetylase (HDAC) inhibitor TSA increases H3 acetylation and enhances fear memory consolidation; that is, long-term memory (LTM) is enhanced, while short-term memory (STM) is unaffected. Conversely, intra-LA infusion of the DNA methyltransferase (DNMT) inhibitor 5-AZA impairs fear memory consolidation. Further, intra-LA infusion of 5-AZA was observed to impair training-related increases in H3 acetylation, and pre-treatment with TSA was observed to rescue the memory consolidation deficit induced by 5-AZA. In our final series of experiments, we show that bath application of either 5-AZA or TSA to amygdala slices results in significant impairment or enhancement, respectively, of long-term potentiation (LTP) at both thalamic and cortical inputs to the LA. Further, the deficit in LTP following treatment with 5-AZA was observed to be rescued at both inputs by co-application of TSA. Collectively, these findings provide strong support that histone acetylation and DNA methylation work in concert to regulate memory consolidation of auditory fear conditioning and associated synaptic plasticity in the LA.

## Introduction

Traditional views of memory formation have emphasized the importance of NMDA receptor (NMDAR)-driven alterations in protein kinase signaling cascades, the activation of transcription factors, and associated changes in gene expression that are thought to be critical for long-term memory and synaptic plasticity [1], [2]. Pavlovian fear conditioning, for example, is known to involve NMDAR-driven alterations in synaptic transmission within the lateral nucleus of the amygdala (LA) [3], [4] and the resultant activation of protein kinase signaling pathways [5], [6], [7], transcription factors [8], and the expression of early and late response genes [9], [10], [11], [12], [13] in LA neurons.

Within the last decade, it has become increasingly clear that epigenetic mechanisms, including modifications of chromatin structure and DNA methylation, play an additional critical role in transcriptional regulation, synaptic plasticity, and memory formation [14], [15], [16], [17]. Chromatin, which consists of DNA packaged tightly around a core of eight histones, is known to be post-translationally regulated by acetylation of histones on their N-terminal tails via histone acetyltransferases (HATs). This process causes chromatin structure to relax, leading to enhanced transcription, and can be reversed by histone deacetylases (HDACs) [18], [19], [20]. Conversely, DNA methylation has typically been associated with transcriptional repression, a process which is catalyzed by DNA methyltransferases (DNMTs) [21]. Both histone acetylation and DNA methylation have been widely implicated in hippocampal-dependent synaptic plasticity and memory formation. Contextual fear conditioning, for example, has been shown to increase acetylation of histone H3 in the hippocampus [22], [23], [24]. Further, HDAC inhibition in the hippocampus has been shown to enhance both synaptic plasticity in area CA1 [23], [24] and hippocampal-dependent memory formation, including object recognition [25] and contextual fear memory [24]. Conversely, intra-hippocampal DNMT inhibition has been shown to impair contextual fear memory [23], [26] and synaptic plasticity in area CA1 [23], [27].

While studies have pointed to a clear and vital role for epigenetic alterations in hippocampal-dependent memory formation, few studies have systematically examined the role of epigenetic mechanisms in amygdala-dependent memory formation [28], [29]. In the present study, we asked whether histone acetylation and DNA methylation are critical for auditory Pavlovian fear conditioning and associated synaptic plasticity in the LA. We first show that acetylation of histone H3 and DNMT3A expression is regulated in an associative manner in LA neurons after fear conditioning. Next, we show that pharmacological manipulation of histone acetylation or DNA methylation in the LA enhances or impairs, respectively, memory consolidation of auditory fear conditioning and long-term potentiation (LTP) at thalamic and cortical inputs to the LA.

## Results

### Auditory fear conditioning regulates histone acetylation and DNMT expression in the LA

Epigenetic mechanisms, including histone acetylation and DNA methylation, have been widely implicated in memory formation, primarily hippocampal-dependent memory tasks such as contextual fear conditioning and object recognition [23], [24], [25], [30]. In this first series of experiments, we asked whether auditory Pavlovian fear conditioning regulates histone acetylation and DNMT expression in the LA, and whether training-related regulation of histone acetylation and DNMT expression is downstream of ERK/MAPK signaling in LA neurons.

#### Auditory Pavlovian fear conditioning regulates acetylation of histone H3 and DNMT3A expression in the LA

**Figure 1:**
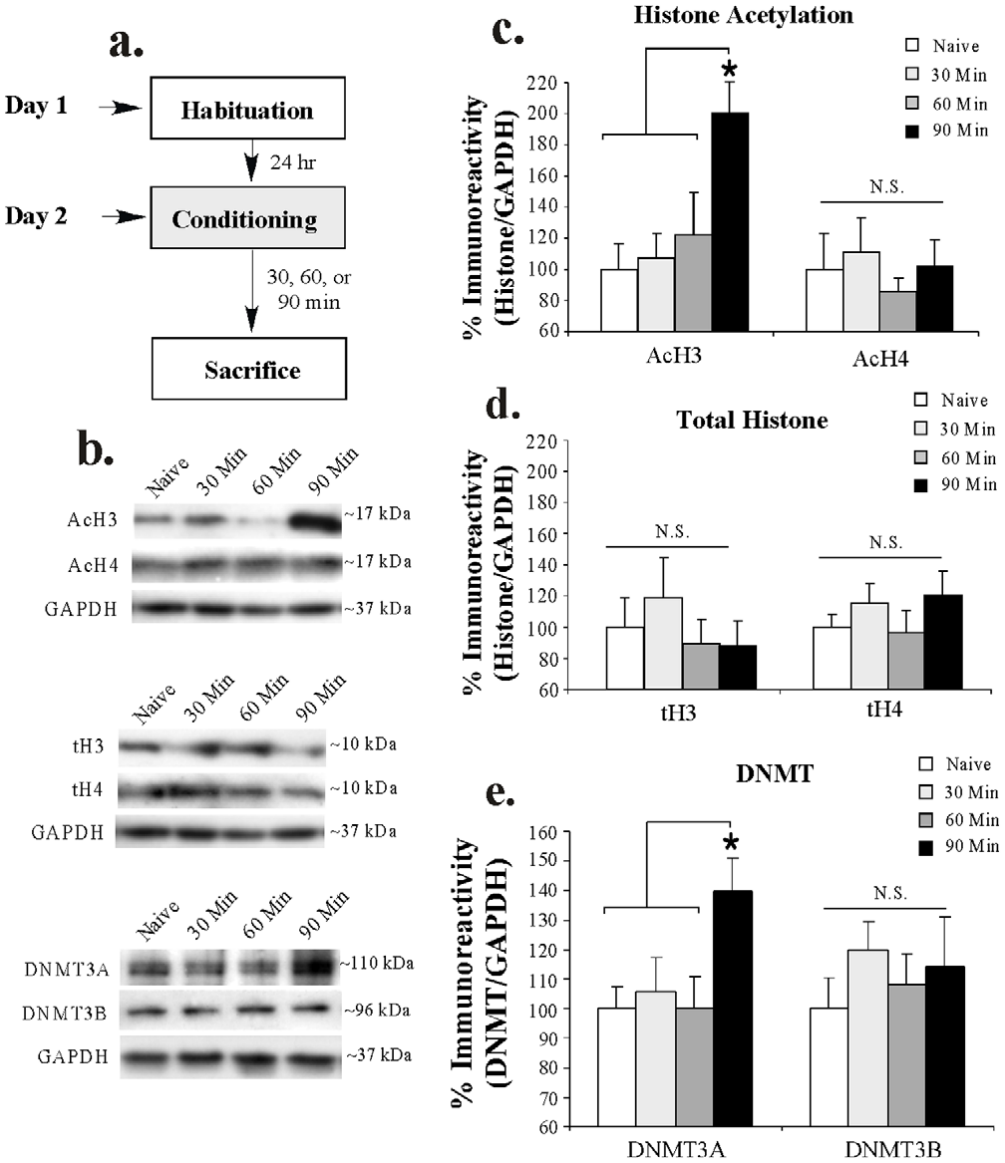
Auditory fear conditioning regulates histone acetylation and DNMT expression in the LA. (A) Schematic of the behavioral protocol. Rats were habituated to handling, trained with 3 tone-shock pairings, and sacrificed at 30, 60, or 90 min later. (*B*) Representative Western blots for acetylated histone (top), total histone (middle), and DNMT expression (bottom) at each time point. (*C*) Mean (±SEM) acetyl-H3 and acetyl-H4 immunoreactivity from LA punches taken from Naïve (n = 7) and trained rats sacrificed at 30 min (n = 8), 60 min (n = 8), or 90 min (n = 7). Here, acetyl-H3 and acetyl-H4 protein levels have been normalized to GAPDH levels for each sample and expressed as a percentage of the Naïve group. (*) *p*<0.05 relative to all other time points. (*D*) Mean (±SEM) total-H3 and total-H4 immunoreactivity from LA punches taken from the samples in (B). Here, total-H3 and total-H4 protein levels have been normalized to GAPDH levels for each sample and expressed as a percentage of the Naïve group. (E) Mean (±SEM) DNMT3A/3B immunoreactivity from LA punches taken from Naïve (n = 7) and trained rats sacrificed at 30 min (n = 8), 60 min (n = 7), or 90 min (n = 8). (*) *p*<0.05 relative to all other time points. Here, DNMT3A/3B protein levels have been normalized to GAPDH levels for each sample and expressed as a percentage of the Naïve group.

In our first series of experiments, we examined changes in histone acetylation and DNMT expression in the LA following auditory fear conditioning using Western blotting (Figure 1). Rats were trained with 3 pairings of a 5 kHz, 75 dB, 20 sec tone that co-terminated with a 1 sec, 1 mA foot shock and sacrificed at either 30, 60, or 90 min following conditioning (Figure 1a). A naïve group received no stimulation and was sacrificed on the same day as the other groups.

Representative Western blots can be viewed in Figure 1b. Western blot analysis on punches taken from the LA revealed that fear conditioning promoted significant increases in histone H3 acetylation [F_(3,26)_ = 4.73, *p*<0.01], while levels of histone H4 acetylation remained unchanged [F_(3,26)_  = 0.21, *p*>0.05; Figure 1c). Post-hoc analysis (Duncan's t-tests) revealed that histone H3 acetylation in the 90 min group differed significantly from that of naïve controls (*p*<0.05) and both the 30 min (*p*<0.05) and 60 min groups (*p*<0.05). Importantly, there were no differences detected in total levels of H3 [F_(3,26)_  = 0.55, *p*>0.05] or H4 [F_(3,27)_  = 0.69, *p*>0.05] at any time point (Figure 1d), indicating the increase in H3 acetylation observed at 90 min was not the result of an overall increase in H3 protein in the LA. Further, the levels of the loading control GAPDH did not differ between groups [F_(3,26)_  = 1.06, *p*>0.05] suggesting an equal amount of protein was loaded within each group.

Analysis of DNMT protein expression revealed a significant increase in DNMT3A [F_(3,26)_  = 3.45, *p*<0.01], while levels of DNMT3B remained unchanged [F_(3,26)_  = 0.47, p>0.05; Figure 1e). Post-hoc analysis revealed that DNMT3A expression in the 90 min group differed significantly from that of naïve controls (*p*<0.05) and both the 30 min (*p*<0.05) and 60 min groups (*p*<0.05). Importantly, the levels of the loading control GAPDH did not differ between groups [F_(3,26)_  = 0.10, *p*>0.05].

#### Auditory fear conditioning, but not immediate shock or tone alone, regulates histone H3 acetylation and DNMT3A expression in the LA

**Figure 2:**
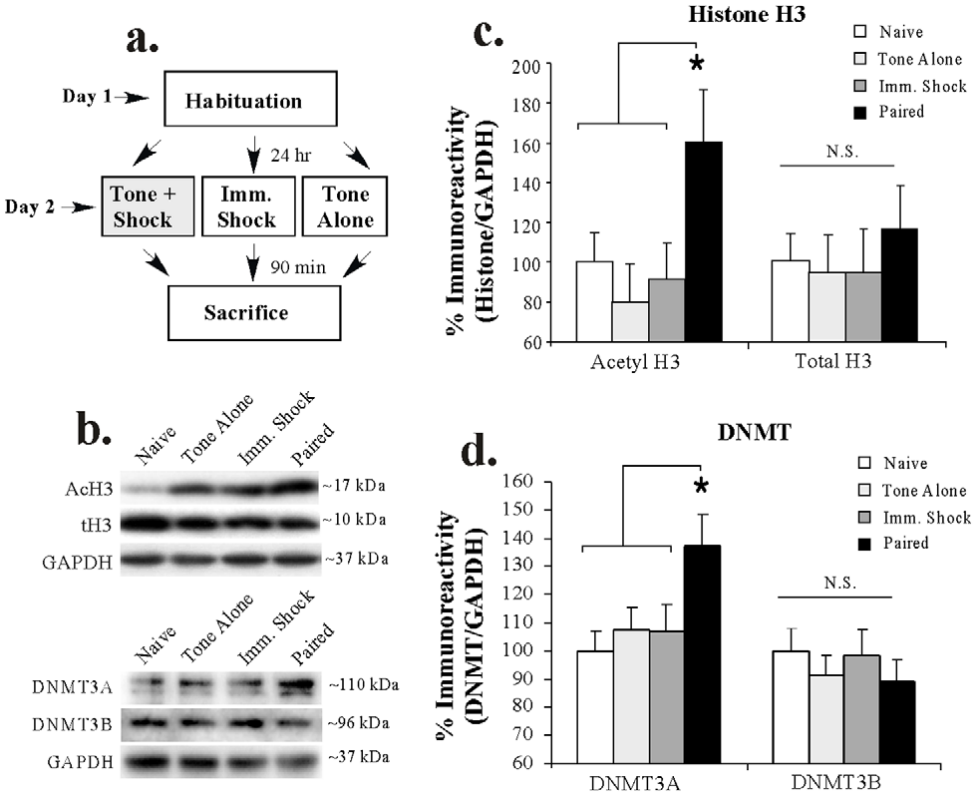
Training-related regulation of histone H3 acetylation and DNMT3A expression in the LA is specific to tone-shock pairing. (A) Schematic of the behavioral protocol. In two separate experiments, rats were either given no stimulation (“Naïve”), 3 tone-alone presentations (“Tone Alone”), 3 immediate shocks (“Imm. Shock”), or 3 tone-shock pairings (“Paired”) and sacrificed 90 min later. (*B*) Representative Western blots for acetylated and total histone H3 (top) and DNMT3A/B expression (bottom) in each group. (*C*) Mean (±SEM) acetyl-H3 and total H3 immunoreactivity from LA punches taken from Naïve (n = 8), Tone Alone (n = 7), Imm. Shock (n = 8), and Paired (n = 6) rats. Here, acetyl-H3 and total H3 protein levels have been normalized to GAPDH levels for each sample and expressed as a percentage of the Naïve group. (*) *p*<0.05 relative to all other groups. (*D*) Mean (±SEM) DNMT3A/B immunoreactivity from LA punches taken from Naïve (n = 8), Tone Alone (n = 8), Imm. Shock (n = 8), and Paired (n = 8) rats. Here, DNMT3A/B protein levels have been normalized to GAPDH levels for each sample and expressed as a percentage of the Naïve group. (*) *p*<0.05 relative to all other groups.

In our next series of experiments, we used Western blotting to ask whether the increase in H3 acetylation and DNMT3A expression in the LA at 90 min after fear conditioning is the result of the tone-shock pairing rather than to presentation of either tone or shock alone. Rats were presented with either 3 tone-shock pairings (“Paired”), 3 tones in the absence of shock (“Tone Alone”), or 3 immediate shocks of equivalent duration and intensity to that of the Paired group (“Imm Shock”). Ninety min later, rats in each group were sacrificed and punches were taken from the LA (Figure 2a). A naïve (“Naïve”) group received no stimulation and was sacrificed on the same day as the other groups. Representative Western blots can be viewed in Figure 2b, while analysis of H3 acetylation can be viewed in Figure 2c. The ANOVA revealed a significant effect for group [F_(3,24)_  = 3.18, p<0.04], with H3 acetylation in the Paired group differing significantly from that of Tone Alone, Imm Shock, and Naïve groups (p<0.05; Duncan's test). No differences were observed between any of the other groups (p>0.05). Further, total levels of histone H3 [F_(3,24)_  = 0.25, p>0.05; Figure 2c] or the loading control GAPDH [F_(3,25)_  = 1.83, p>0.05; not shown] did not differ between the groups.

Western blot analysis of DNMT3A expression can be viewed in Figure 2d. The ANOVA for DNMT3A revealed a significant effect for group [F_(3,28)_  = 3.25, *p*<0.05], with DNMT3A expression in the Paired group differing significantly from that of Tone Alone, Imm Shock, and Naïve groups (*p*<0.05; Duncan's test). No differences were observed between any of the other groups (*p*>0.05). No significant regulation of DNMT3B expression was observed [F_(3,28)_  = 0.42, *p*>0.05]. Further, levels of the loading control GAPDH did not differ between the groups [F_(3,28)_  = 0.82, *p*>0.05; not shown].

#### Training-related regulation of histone H3 acetylation and DNMT3A expression in the LA is downstream of ERK/MAPK activation

**Figure 3:**
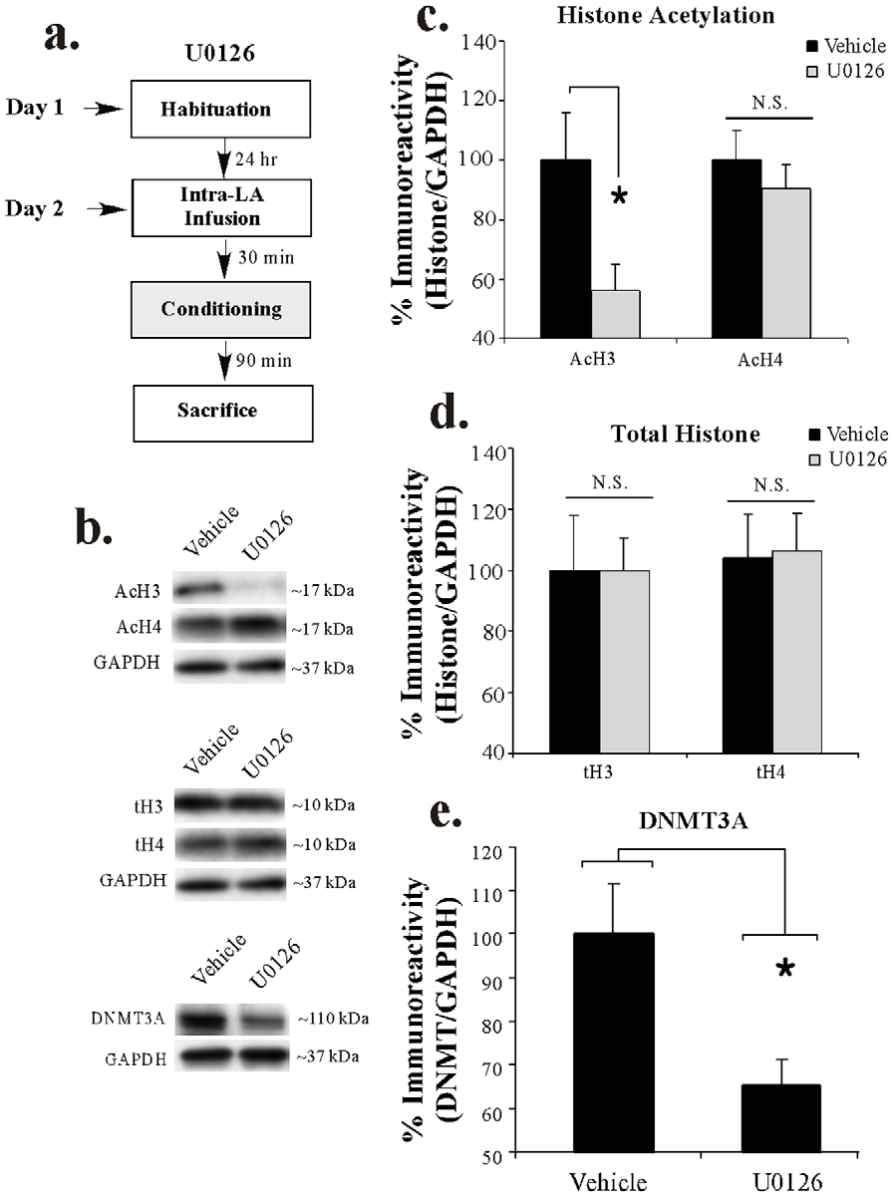
Regulation of histone H3 acetylation and DNMT3A expression in the LA following fear conditioning is ERK-dependent. (A) Schematic of the behavioral protocol. Rats received intra-LA infusion of either vehicle (n = 7) or U0126 (n = 8) followed 30 min later by fear conditioning consisting of 3 tone-shock pairings and were sacrificed 90 min after training. (*B*) Representative Western blots for acetylated histone (top), total histone (middle), and DNMT3A expression (bottom) at each time point. (*C*) Mean (±SEM) acetyl-H3 and acetyl-H4 immunoreactivity from punches taken from the LA. Here, acetyl-H3 and acetyl-H4 protein levels have been normalized to GAPDH levels for each sample and expressed as a percentage of the vehicle-infused group. (*) *p*<0.05 relative to vehicle group. (*D*) Mean (±SEM) total-H3 and total-H4 immunoreactivity from the samples in (C). Here, total-H3 and total-H4 protein levels have been normalized to GAPDH levels for each sample and expressed as a percentage of the vehicle-infused group. (*E*) Mean (±SEM) DNMT3A immunoreactivity from punches taken from the LA from vehicle (n = 7) and U0126-treated rats (n = 7). Here, DNMT3A protein levels have been normalized to GAPDH levels for each sample and expressed as a percentage of the vehicle-infused group. (*) *p*<0.05 relative to vehicle group.

We next asked whether the training-related regulation of histone H3 and DNMT3A expression in the LA is downstream of ERK/MAPK signaling. Previous studies have shown that auditory fear conditioning regulates ERK1/2 activation in the LA, with peaks at both 5 and 60 min following training [5], [31]. Here, rats received intra-LA infusion of either the MEK inhibitor U0126 (1 µg/side) or vehicle 30 min prior to fear conditioning consisting of 3 pairings of a 5 kHz, 75 dB, 20 sec tone that co-terminated with a 1 sec, 1 mA foot shock. Rats were then sacrificed 90 min following conditioning and punches from the LA were taken and processed for H3/H4 acetylation and DNMT3A expression using Western blotting (Figure 3a). Representative blots can be viewed in Figure 3b. Infusion of U0126 was observed to significantly impair the training-related increase in H3 acetylation [t_(13)_  = 2.37, *p*<0.04] in the LA, while levels of H4 acetylation remained unchanged [t_(13)_  = 0.73, *p*>0.05; Figure 3c]. Importantly, levels of total H3 [t_(13)_  = 0.17, *p*>0.05] and H4 [t_(13)_  = 0.38, *p*>0.05] remained unchanged following U0126 infusion (Figure 3d), and the loading control GAPDH failed to differ between groups [t_(14)_  = 1.19, *p*>0.05]. Similarly, treatment with U0126 was observed to significantly impair the training-related increase in DNMT3A expression in the LA [t_(12)_  = 2.66, *p*<0.03], while levels of the loading control GAPDH did not differ between groups [t_(12)_  = 1.18, *p*>0.05].

Thus, amygdala-dependent auditory Pavlovian fear conditioning regulates histone H3 acetylation and DNMT3A expression in the LA in an ERK-dependent manner. Further, this training-related increase in H3 acetylation and DNMT3A expression is the result of the associative pairing of tone and shock rather than to presentation of either stimulus alone.

### Intra-LA infusion of an HDAC inhibitor enhances fear memory consolidation

In our initial series of experiments we showed that auditory fear conditioning regulates H3 acetylation. In our next series of experiments, we asked whether this training-related increase in H3 acetylation is required for the formation and/or consolidation of auditory fear conditioning. Several recent studies have indicated that intra-CA1 infusion of HDAC inhibitors increases H3 acetylation in the hippocampus and enhances consolidation of a contextual fear memory [22], [24]. The effect of HDAC inhibition in amygdala-dependent fear memory consolidation, however, has received relatively little attention. One recent study reported enhanced consolidation of an auditory fear memory following systemic injection of the HDAC inhibitor valproic acid [29]. Further, intra-LA infusion of the HDAC inhibitor TSA has been shown to enhance fear memory consolidation to a visual CS using the fear-potentiated startle paradigm [28]. Here, we systemically examined the effects of intra-LA infusion of an HDAC inhibitor on the consolidation of auditory Pavlovian fear conditioning.

#### HDAC inhibition increases acetylation of histone H3 in the LA following auditory fear conditioning

**Figure 4:**
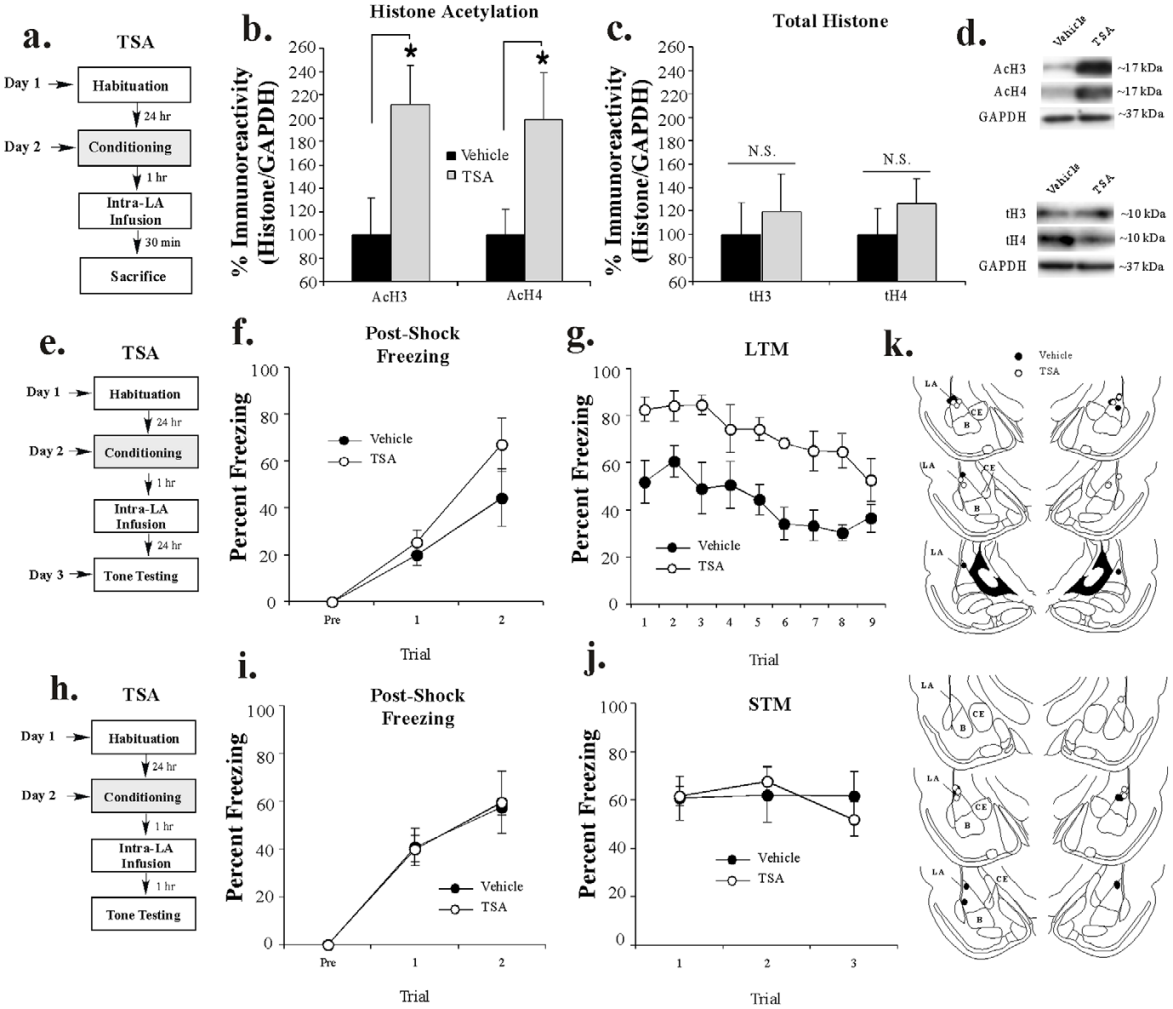
Intra-LA infusion of an HDAC inhibitor increases histone acetylation and enhances auditory fear memory consolidation. (A) Schematic of the behavioral protocol. Rats were trained with 2 tone-shock pairings followed 1 hr later by intra-LA infusion of either vehicle (n = 8) or TSA (n = 8) and sacrificed 30 min after infusion. (*B*) Mean (±SEM) acetyl-H3 and acetyl-H4 immunoreactivity from punches taken from the LA. Here, acetyl-H3/H4 protein levels have been normalized to GAPDH levels for each sample and expressed as a percentage of the vehicle-infused group. (*) *p*<0.05 relative to vehicle-infused rats. (*C*) Mean (±SEM) total-H3/H4 from the samples in (B). Here, total-H3/H4 protein levels have been normalized to GAPDH levels for each sample and expressed as a percentage of the vehicle group. (*D*) Representative blots for acetyl-H3/H4 and total-H3/H4, respectively. (*E*) Schematic of behavioral protocol. Rats were conditioned with 2 tone-shock pairings, followed 1 hr later by intra-LA infusion of vehicle (n = 6) or TSA (n = 6). LTM was assessed ∼24 hr after training in a distinct context. (*F*) Mean (±SEM) post-shock freezing scores in each group following each conditioning trial. (*G*) Mean (±SEM) LTM retention test scores across each trial. (*H*) Schematic of behavioral protocol. Rats were conditioned with 2 tone-shock pairings, followed 1 hr later by intra-LA infusion of vehicle (n = 4) or TSA (n = 4). STM was assessed 1 hr after infusion in a distinct context. (*I*) Mean (±SEM) post-shock freezing scores in each group following each conditioning trial. (*J*) Mean (±SEM) STM retention test scores across each trial. (*K*) Histological verification of cannula placements for rats infused with vehicle (black circles) or TSA (white circles) for the LTM (top) and STM (bottom) experiments. Panels adapted from Paxinos and Watson [40].

In our first experiment, we determined whether intra-LA infusion of TSA results in enhanced histone acetylation in the LA. Rats were trained with 2 tone-shock pairings followed 1 hr later by intra-LA infusion of vehicle or TSA (1 µg/side). This slightly weaker training protocol (2, rather than 3, tone-shock pairings) was used to avoid a ceiling effect in freezing which may have prevented our ability to detect a memory enhancing effect of TSA. Thirty min following infusion (90 min after training) rats were sacrificed and punches were taken from the LA (Figure 4a). Western blotting revealed a significant increase in both H3 [t_(14)_  = 2.44, *p*<0.03] and H4 [t_(14)_  = 2.20, *p*<0.05] acetylation in TSA-infused rats relative to vehicle-infused controls (Figure 4b). Importantly, levels of total H3 [t_(14)_  = 0.45, *p*>0.05] or H4 [t_(14)_  = 0.86, *p*>0.05] did not differ between the two groups (Figure 4c). Further, levels of the loading control GAPDH failed to differ between the two groups [H3: t_(14)_  = 0.13, *p*>0.05; H4: t_(14)_  = 0.29, *p*>0.05; not shown]. Representative blots can be viewed in Figure 4d.

#### HDAC inhibition in the LA enhances auditory fear memory consolidation

To examine the effect of intra-LA HDAC inhibition on fear memory consolidation, rats were trained with 2 tone-shock pairings as above followed 1 hr later by intra-LA infusion of either vehicle or TSA (1 µg/side; Figure 4e). No differences were observed in post-shock freezing during training (Figure 4f). The ANOVA for PSF scores revealed nonsignificant effects for group [F_(1,7)_  = 1.52, *p*>0.05] and the group by trial interaction [F_(2,14)_  = 1.13, *p*>0.05], but a significant effect for trial [F_(2,14)_  = 25.83, *p*<0.01]. During the LTM test, however, TSA-infused rats demonstrated a higher level of fear memory retention relative to vehicle-infused controls (Figure 4g). The ANOVA for LTM scores revealed significant effects for group [F_(1,10)_  = 15.86, *p*<0.002] and trial [F_(8,80)_  = 6.78, *p*<0.001], but a nonsignificant group by trial interaction [F_(8,80)_  = 0.69, *p*>0.05]. Cannula placements are depicted in Figure 4k (top).

Next, we examined the effects of intra-LA infusion of TSA on STM in a separate group of rats. Rats were trained and infused as above and STM was assessed in a distinct context 1 hr following infusion (Figure 4h). No differences in post-shock freezing were observed between groups (Figure 4i). The ANOVA for PSF scores revealed no significant effects for group [F_(1,6)_  = 0.01, *p*>0.05] or the group by trial interaction [F_(2,12)_  = 0.01, *p*>0.05]; however there was a significant main effect for trial [F_(2,12)_  = 44.48, *p*<0.01]. Further, TSA and vehicle-infused rats exhibited comparable levels of freezing during the STM test (Figure 4j). The ANOVA for STM scores revealed nonsignificant effects for group [F_(1,6)_  = 0.01, *p*>0.05], trial [F_(2,12)_  = 1.19, *p*>0.05], and the group by trial interaction [F_(2,12)_  = 1.15, *p*>0.05]. Therefore, TSA does not interfere with acquisition or STM formation of an auditory fear memory in the LA. Cannula placements for rats infused with either vehicle or TSA are depicted in Figure 4k (bottom).

### Intra-LA infusion of a DNMT inhibitor impairs fear memory consolidation

DNA methylation has been shown to negatively regulate memory via its ability to repress gene transcription [17], [30]. Interestingly, however, several recent studies have shown that inhibition of DNMT activity results in deficits in memory consolidation in hippocampal-dependent memory tasks [23], [30]. In our next series of experiments, we examined the effect of intra-LA infusion of the DNMT inhibitor 5-AZA on fear memory consolidation.

#### DNMT inhibition in the LA impairs auditory fear memory consolidation

**Figure 5:**
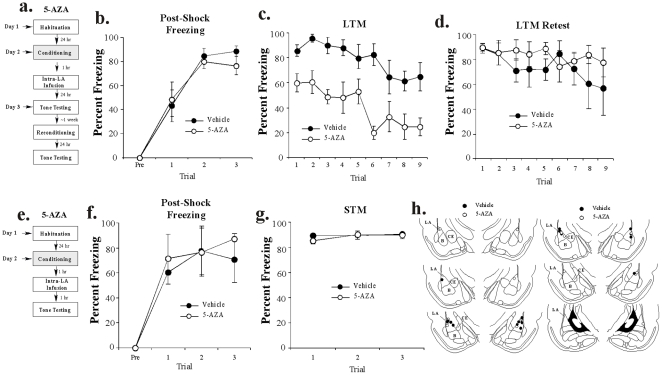
Intra-LA infusion of a DNMT inhibitor impairs auditory fear memory consolidation. (A) Schematic of behavioral protocol. Rats were conditioned with 3 tone-shock pairings, followed by intra-LA infusion of vehicle or 5-AZA 1 hr later (n = 5, each group). LTM was assessed ∼24 hr after training in a distinct context. Rats were re-conditioned drug free and re-tested for LTM ∼1 week later. (*B*) Mean (±SEM) post-shock freezing scores in each group following each conditioning trial. (*C*) Mean (±SEM) LTM retention test scores across each trial. (*D*) Mean (±SEM) LTM retention re-test scores across each trial following re-conditioning one week later. (*E*) Schematic of behavioral protocol. Rats were conditioned with 3 tone-shock pairings, followed 1 hr later by intra-LA infusion of vehicle (n = 4) or 5-AZA (n = 4). STM was assessed 1 hr after training in a distinct context. (*F*) Mean (±SEM) post-shock freezing scores in each group following each conditioning trial. (*G*) Mean (±SEM) STM retention test scores across each trial. (*H*) Histological verification of cannula placements for rats in LTM (left) and STM (right) experiments infused with vehicle (black circles) or 5-AZA (white circles). Panels adapted from Paxinos and Watson [40].

Rats were trained using 3 tone-shock pairings followed 1 hr later by intra-LA infusion of vehicle or 5-AZA (1 µg/side). Long-term memory was tested 24 hrs later in a distinct context (Figure 5a). No differences were observed in levels of post-shock freezing between 5-AZA and vehicle-infused rats on any trial (Figure 5b). The ANOVA for PSF scores revealed no significant effects for group [F_(1,8)_  = 0.22, *p*>0.05] or the group by trial interaction [F_(3,24)_  = 0.38, *p*>0.05]; however, there was a significant main effect of trial [F_(3,24)_  = 43.41, *p*<0.01]. The results of the LTM test are depicted in Figure 5c. The ANOVA revealed significant main effects for group [F_(1,8)_  = 22.15, *p*<0.001] and trial [F_(8,64)_  = 5.64, *p*<0.001], but a nonsignificant group by trial interaction [F_(8,64)_  = 0.94, *p*>0.05]. Cannula placements for rats in this experiment are depicted in Figure 5h (left).

To ensure that 5-AZA did not have non-specific effects or cause damage to the amygdala, we retrained and retested the groups that received vehicle or 5-AZA drug-free one week later. As before, we observed no significant differences in post-shock freezing (not shown). The ANOVA for PSF scores in the re-trained groups revealed nonsignificant effects for group [F_(1,8)_  = 3.03, *p*>0.05] and the group by trial interaction [F_(3,24)_  = 1.02, *p*>0.05], but there was a significant effect for trial [F_(3,24)_  = 26.08, *p*<0.01]. Further, when long-term memory was assessed ∼24 hrs later, no significant differences were observed (Figure 5d). The ANOVA for the LTM re-test scores revealed no significant effects of group [F_(1,8)_  = 1.00, *p*>0.05], trial [F_(8,64)_  = 0.91, *p*>0.05], or the group by trial interaction [F_(8,64)_  = 0.66, *p*>0.05], indicating that rats in the 5-AZA-infused group were in fact still able to learn at levels equivalent to that of the vehicle-infused group.

Next, we examined the effect of intra-LA infusion of 5-AZA on STM. A separate group of rats was trained and infused as above, and STM was assessed in a distinct context 1 hr following infusion (2 hrs following training; Figure 5e). No differences in post-shock freezing were observed between groups (Figure 5f). The ANOVA for PSF scores revealed no significant effects for group [F_(1,6)_  = 0.64, *p*>0.05] or the group by trial interaction [F_(3,18)_  = 0.29, *p*>0.05]; however there was a significant main effect for trial [F_(3,18)_  = 9.34, *p*<0.01]. Further, freezing between 5-AZA and vehicle-infused rats was comparable during the STM test (Figure 5g). The ANOVA for STM scores revealed nonsignificant main effects for group [F_(1,6)_  = 0.03, *p*>0.05], trial [F_(2,12)_  = 0.96, *p*>0.05], and the group by trial interaction [F_(2,12)_  = 0.87, *p*>0.05]. Therefore, 5-AZA does not interfere with acquisition or STM formation of an auditory fear memory in the LA. Cannula placements for the STM experiment are shown in Figure 5h (right).

Collectively, our findings suggest that modifications in both histone acetylation and DNA methylation are critical for memory consolidation of auditory Pavlovian fear conditioning in the LA; in both cases, LTM is affected, while acquisition and STM are not.

### Histone acetylation and DNA methylation work in concert to regulate memory consolidation in the LA

Thus far, we have shown that auditory fear conditioning regulates histone H3 acetylation and DNMT3A expression in the LA, and that pharmacological manipulation of HDAC or DNMT activity in the LA enhances or impairs, respectively consolidation of an auditory fear memory. Recent studies have suggested that one way in which DNMT inhibitors may negatively regulate memory formation is by influencing histone acetylation [23]. For example, intra-CA1 infusion of a DNMT inhibitor has been shown to impair both contextual fear memory and the training-related increase in histone H3 acetylation in the hippocampus [23]. Further, pre-treatment with an HDAC inhibitor has been shown to rescue the memory deficit induced by a DNMT inhibitor [23]. In this series of experiments, we first examined whether intra-LA infusion of the DNMT inhibitor 5-AZA impairs training-related increases in histone H3 acetylation. Next, we asked whether these two epigenetic mechanisms work in concert during auditory fear memory consolidation by examining whether pre-treatment with TSA can rescue the 5-AZA-induced consolidation deficit in the LA.

#### DNMT inhibition impairs training-related histone H3 acetylation in the LA

**Figure 6:**
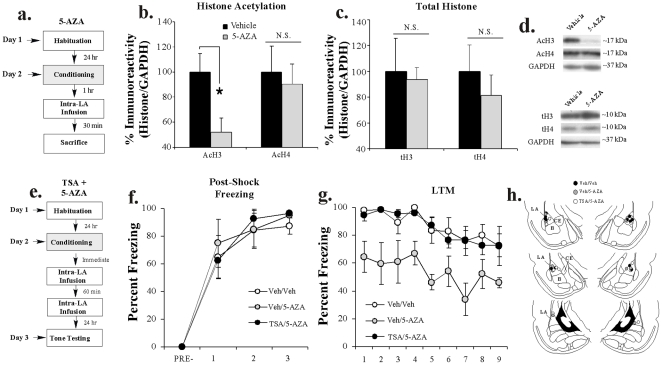
Histone acetylation and DNA methylation interact to regulate memory consolidation in the LA. (A) Schematic of the behavioral protocol. Rats were trained with 3 tone-shock pairings followed 1 hr later by intra-LA infusion of either vehicle (n = 7) or 5-AZA (n = 7) and sacrificed 30 min after infusion. *(B)* Mean (±SEM) acetyl-H3 and acetyl-H4 immunoreactivity from punches taken from the LA. Here, acetyl-H3 and acetyl-H4 protein levels have been normalized to GAPDH levels for each sample and expressed as a percentage of the vehicle group. (*) *p*<0.05 relative to vehicle group. (*C*) Mean (±SEM) total-H3 and total-H4 immunoreactivity from the samples in (B). Here, total-H3 and total-H4 protein levels have been normalized to GAPDH levels for each sample and expressed as a percentage of the vehicle-infused group. (*D*) Representative blots for acetyl-H3/H4 and total-H3/H4, respectively. (*E*) Schematic of the behavioral protocol. Rats were trained and immediately after given intra-LA infusion of either (1 µg in 0.5 µl/side) TSA or Vehicle (0.5 µl/side) followed 60 min later by intra-LA infusion of (1 µg in 0.5 µl/side) 5-AZA or Vehicle (0.5 µl/side), creating the following groups: Veh-Veh (*n* = 6), Veh-5-AZA (*n* = 5), and TSA-5-AZA (*n* = 6). LTM was examined 24 hrs later. (*F*) Post-shock freezing scores in each group immediately after the conditioning trials. (*G*) Mean (±SEM) LTM retention test scores across each trial. (*H*) Histological verification of cannula placements for rats infused with Vehicle-Vehicle (black circles), or Vehicle-5-AZA (gray circles), or TSA-5-AZA (white circles). Panels adapted from Paxinos and Watson [40].

In our first experiment, rats were trained with 3 tone-shock pairings followed 1 hr later by intra-LA infusion of vehicle or 5-AZA (1 µg/side). Thirty min following infusion (90 min following training), rats were sacrificed and punches were taken from the LA (Figure 6a). Western blotting on protein lysates taken from around the cannula tips showed a significant decrease in H3 [t_(12)_  = 2.54, *p*<0.02], but not H4 [t_(12)_  = 0.35, *p*>0.05] acetylation in the 5-AZA-infused group (Figure 6b). Representative blots can be viewed in Figure 6d. Importantly, levels of total H3 [t_(12)_  = 0.16, *p*>0.05] or H4 [t_(12)_  = 0.68, *p*>0.05] did not differ between the two groups (Figure 6c,d). Additionally, levels of the loading control GAPDH failed to differ between the two groups [H3: t_(13)_  = 0.40, *p*>0.05; H4: t_(13)_  = 0.78, *p*>0.05; not shown].

#### Inhibition of HDAC activity in the LA rescues the consolidation deficit induced by a DNMT inhibitor

In our second experiment, rats were trained with three tone-shock pairings and immediately infused with either TSA (1 µg/0.5 µl) or vehicle (0.5 µl/side) followed 1 hr later by infusion of either 5-AZA (1 µg/0.5 µl) or vehicle (0.5 µl/side). All rats were given a LTM test 24 hrs later (Figure 6e). There was no difference in the levels of post-shock freezing between the vehicle/vehicle, vehicle/5-AZA and TSA/5-AZA groups (Figure 6f). An ANOVA (group by trial) revealed a significant main effect of trial [*F*_(3,42)_  = 71.02, *p*<0.01], but no significant main effect of group [*F*_(2,14)_  = 0.12, *p*>0.05] or group by trial interaction [*F*_(6,42)_  = 0.28, *p*>0.05]. Further, on the next day, the group infused with vehicle/5-AZA exhibited impaired LTM, while the TSA/vehicle and vehicle/vehicle infused groups showed equivalent levels of intact LTM (Figure 6g). The ANOVA (group by trial) revealed significant main effects of group [*F*_(2,14)_  = 8.20, *p*<0.01] and trial [*F*_(8,112)_  = 6.82, *p*<0.01], but no significant group by trial interaction [*F*_(12,112)_  = 0.44, *p*>0.05]. Thus, these findings both confirm that intra-LA infusion of a DNMT inhibitor interferes with LTM formation, and also indicate that pre-treatment with an HDAC inhibitor can rescue the consolidation deficit induced by a DNMT inhibitor. Cannula placements are shown in Figure 6h.

### DNMT and HDAC inhibition impairs or enhances, respectively, LTP at thalamic and cortical inputs to the LA

In our final series of experiments, we examined whether histone acetylation and DNA methylation are involved in synaptic plasticity in the LA. We first examined the effects of DNMT and HDAC inhibition on LTP at thalamic and cortical inputs to the LA. Next, we examined whether co-application of an HDAC inhibitor can rescue the DNMT inhibitor-induced impairment in LTP in amygdala slices.

#### Bath application of a DNMT inhibitor impairs LTP at thalamic and cortical inputs to the LA

**Figure 7:**
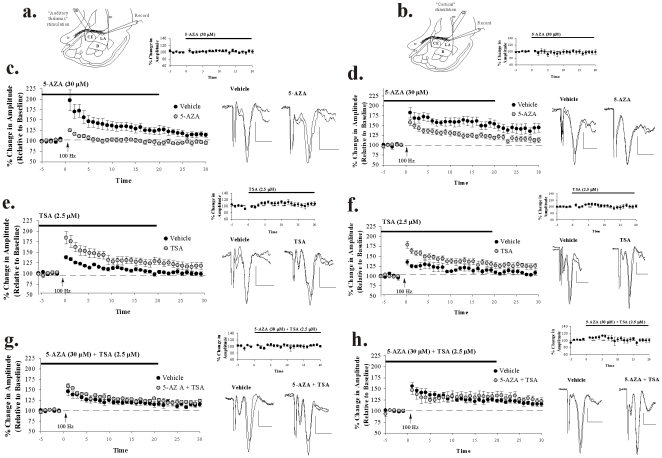
DNMT and HDAC inhibition impairs or enhances, respectively, amygdala LTP at thalamic and cortical inputs. (A–B) Schematic of amygdala slice preparation for “thalamic” and “cortical” stimulation experiments, showing placement of stimulating and recording electrodes. (*C*) Mean (±SEM) percent field potential amplitude (relative to baseline) in slices treated with vehicle (n = 7; black circles) or 30 µM 5-AZA (n = 5; gray circles) followed by LTP induction at thalamic inputs. Traces from an individual experiment before and 30 min following tetanic stimulation and baseline transmission following 20 min of bath application of drug are shown in the *inset*. Scale, 0.2 mV by 10 msec. (*D*) Mean (±SEM) percent field potential amplitude (relative to baseline) in slices treated with vehicle (n = 6; black circles) or 30 µM 5-AZA (n = 7; gray circles) followed by LTP induction at cortical inputs. Traces from an individual experiment before and 30 min following tetanic stimulation and baseline transmission following 20 min of bath application of drug are shown in the *inset*. Scale, 0.2 mV by 10 msec. (*E*) Mean (±SEM) percent field potential amplitude (relative to baseline) in slices treated with vehicle (n = 10; black circles) or 2.5 µM TSA (n = 7; gray circles) followed by LTP at thalamic inputs. Traces from an individual experiment before and 30 min following tetanic stimulation and transmission following 20 min of bath application of drug are shown in the *inset*. Scale, 0.2 mV by 10 msec. (*F*) Mean (±SEM) percent field potential amplitude (relative to baseline) in slices treated with vehicle (n = 6; black circles) or 2.5 µM TSA (n = 7; gray circles) followed by LTP at cortical inputs. Traces from an individual experiment before and 30 min following tetanic stimulation and transmission following 20 min of bath application of drug are shown in the *inset*. Scale, 0.2 mV by 10 msec. (*G*) Mean (±SEM) percent field potential amplitude (relative to baseline) in slices treated with vehicle (n = 6; black circles) or 30 µM 5-AZA+2.5 µM TSA (n = 6; gray circles) followed by LTP at thalamic inputs. Traces from an individual experiment before and 30 min following tetanic stimulation and transmission following 20 min of bath application of drug are shown in the *inset*. Scale, 0.2 mV by 10 msec. (*H*) Mean (±SEM) percent field potential amplitude (relative to baseline) in slices treated with vehicle (n = 6; black circles) or 30 µM 5-AZA+2.5 µM TSA (n = 5; gray circles) followed by LTP at cortical inputs. Traces from an individual experiment before and 30 min following tetanic stimulation and transmission following 20 min of bath application of drug are shown in the *inset*. Scale, 0.2 mV by 10 msec.

In our first series of experiments, we examined the effects of bath application of a DNMT inhibitor on LTP at thalamic (Figure 7a) and cortical (Figure 7b) inputs to the LA, *in vitro*. Bath application of 5-AZA (30 µM) significantly impaired LTP at both inputs (Figure 7c-d). In our thalamic LTP experiments, control slices showed 117.60±6.86% potentiation, which differed significantly from baseline [t_(6)_  = 2.73, *p*<0.03]. In contrast, slices treated with 5-AZA exhibited impaired LTP, potentiating to only 88.37±5.58% which did not differ significantly from baseline [t_(4)_  = 1.33, *p*>0.05] but did differ significantly from ACSF controls [t_(10)_  = 2.48, *p*<0.03]. In our cortical LTP experiments, control slices showed 141.80±10.52% potentiation, which differed significantly from baseline [t_(5)_  = 4.08, *p*<0.01]. In contrast, slices treated with 5-AZA exhibited impaired LTP, potentiating to 115.51±6.03%, which did not differ significantly from baseline [t_(6)_  = 2.28, *p*>0.05] but did differ significantly from control slices [t_(11)_  = 2.24, *p*<0.04]. Further, bath application of 5-AZA alone had no effect on routine synaptic transmission in either pathway (Figure 7c-d, insets). A comparison of the amplitude of field-evoked potentials before and 20 min following perfusion of 5-AZA (just prior to LTP induction) revealed no significant effect in either the thalamic [t_(8)_  = 0.63, *p*>0.05] or cortical [t_(14)_  = 0.71, *p*>0.05] input pathways.

#### Bath application of an HDAC inhibitor enhances LTP at thalamic and cortical inputs to the LA

We next examined the effects of bath application of the HDAC inhibitor TSA on amygdala LTP, *in vitro*. For this series of experiments, we used a slightly weaker LTP induction protocol that does not induce LTP in controls (see Methods). Using this protocol, we found that bath application of TSA (2.5 µM) significantly enhances LTP at both inputs (Figure 7e-f). In our thalamic LTP experiments, the ACSF-perfused control slices failed to exhibit LTP, potentiating to 100.41±3.09% and failing to differ from baseline [t_(9)_  = 0.28, *p*>0.05]. In contrast, slices treated with TSA exhibited LTP, potentiating to 120.57±7.44% and differing significantly from baseline [t_(6)_  = 2.84, *p*<0.02] and from control slices [t_(15)_  = 2.80, *p*<0.01]. In our cortical LTP experiments, ACSF-infused control slices failed to exhibit LTP, potentiating to 108.53±3.92% and failing to differ from baseline [t_(5)_  = 2.35, *p*>0.05]. In contrast, slices treated with TSA exhibited LTP, potentiating to 126.25±6.28% and differing significantly from baseline [t_(6)_  = 4.30, *p*<0.01] and from control slices [t_(11)_  = 2.29, *p*<0.04]. Further, bath application of TSA alone had no effect on routine synaptic transmission in either the thalamic [t_(12)_  = 1.32, *p*>0.05] or cortical [t_(12)_  = 0.03, *p*>0.05] input pathways (Figure 7e–f, insets).

#### HDAC inhibition rescues the LTP impairment induced by DNMT inhibition at thalamic and cortical inputs to the LA

In our final series of LTP experiments, we asked whether the HDAC inhibitor TSA could rescue the LTP deficit observed following treatment with the DNMT inhibitor 5-AZA at both thalamic and cortical inputs (Figure 7g–h). In our thalamic LTP experiments, slices treated with ACSF alone exhibited significant LTP, potentiating to 114.53±4.23% and differing significantly from baseline [t_(5)_  = 3.16, *p*<0.02]. Slices treated with a combination of the drugs (30 µM 5-AZA+2.5 µM TSA) also exhibited LTP, potentiating to 120.63±4.44% and significantly differing from baseline [t_(5)_  = 4.03, *p*<0.01]. However, the two groups failed to differ from one another [t_(10)_  = 0.99, *p*>0.05] indicating that TSA can rescue LTP in the presence of 5-AZA co-administration. In our cortical LTP experiments, ACSF treated slices exhibited significant LTP, potentiating to 119.28±4.26% and differing significantly from baseline [t_(5)_  = 4.57, *p*<0.01]. Slices treated with a combination of the drugs (30 µM 5-AZA+2.5 µM TSA) also exhibited LTP, potentiating to 128.28±6.88% and significantly differing from baseline [t_(4)_  = 4.14, *p*<0.01] but not from ACSF controls [t_(9)_  = 1.15, *p*>0.05]. Further, co-application of 5-AZA and TSA alone had no effect on routine synaptic transmission in either the thalamic [t_(10)_  = 0.36, *p*>0.05] or cortical [t_(8)_  = 0.24, *p*>0.05] input pathways (Figure 7g–h, insets).

Thus, epigenetic alterations regulate synaptic plasticity in the LA in a qualitatively similar manner to that observed in amygdala-dependent memory consolidation.

## Discussion

Epigenetic mechanisms, including histone modifications and DNA methylation, have recently been shown to play a crucial role in both long-term memory consolidation and associated synaptic plasticity [15], [16], [17]. While these mechanisms have been extensively studied in the hippocampus and in regions of the brain associated with drug addiction [14], [32], [33], relatively little is known about their function in amygdala-dependent learning and memory. In the present study, we systemically examined the role of epigenetic alterations in auditory Pavlovian fear conditioning and associated synaptic plasticity in the LA using a combination of biochemical, behavioral, and electrophysiological techniques. Collectively, our findings indicate that auditory fear conditioning regulates histone H3 acetylation and DNMT3A expression in an ERK-dependent and associative manner in LA neurons. Further, pharmacological manipulation of histone acetylation and DNA methylation in the LA enhances or impairs, respectively, memory consolidation of auditory fear conditioning and associated synaptic plasticity at LA synapses.

Post-translational modifications in chromatin structure via histone acetylation have been widely implicated in cellular differentiation and development, and, more recently, in synaptic plasticity and memory formation [15], [16], [34]. Unmodified chromatin is considered highly inhibitory to transcription as the result of tight binding of histones to DNA via positively charged lysine residues on the N-terminal tails of histone proteins. Acetylation of histones via HATs neutralizes the positive charge on the lysine residue, relaxing the histone-DNA bond and allowing transcription factors to access DNA [18], [19], [20]. Acetylation of Lys-14 on histone H3 appears to be particularly important for transcriptional regulation. Acetylation of histone H3 on Lys-14 has been shown to be regulated in hippocampal-dependent memory tasks, including contextual fear conditioning [23], [24], [25]. Further, treatment with HDAC inhibitors, which prevent de-acetylation, has been shown to enhance memory consolidation for contextual fear conditioning, novel object recognition and LTP in area CA1 [22], [23], [24]. In the present study, we show that auditory Pavlovian fear conditioning leads to a significant increase in the acetylation of histone H3, but not H4, in the LA that is not accounted for by presentation of tone or shock alone. Further, intra-LA infusion of the HDAC inhibitor TSA 1 hr following auditory fear conditioning significantly enhances fear memory consolidation; that is, LTM is enhanced, while STM is unaffected. Finally, bath application of TSA to amygdala slices significantly enhances LTP at thalamic and cortical inputs to the LA. These findings provide strong evidence that modification of chromatin via histone acetylation plays an important role in amygdala-dependent memory consolidation and associated synaptic plasticity.

Our findings suggest that DNA methylation is a second major source of epigenetic modification that is critical for fear memory consolidation and synaptic plasticity in the LA. The methylation of cytosine residues on DNA via DNMTs is typically thought to negatively regulate transcription via preventing the binding of transcription factors [16], [27], [30], [35]. In development, this process has been associated with gene silencing and cellular differentiation, and is believed to be a long-lasting, static process [16], [21]. Neurons, however, are known to express high levels of DNMT mRNA into adulthood, suggesting that dynamic regulation of DNA methylation may be critical for neuronal function, including synaptic plasticity and memory formation. Previous reports have shown, for example, that contextual fear conditioning leads to an increase in the expression of DNMT3A/B mRNA in hippocampal area CA1 [30]. Further, intra-hippocampal infusion or bath application of DNMT inhibitors impairs memory consolidation of contextual fear conditioning and LTP in area CA1 [23], [30]. In our own experiments, we show that auditory fear conditioning regulates the expression of DNMT3A in the LA. Further, intra-LA infusion of the DNMT inhibitor 5-AZA impairs both memory consolidation of auditory fear conditioning and LTP at thalamic and cortical inputs to the LA. While our findings of impaired memory and synaptic plasticity following treatment with 5-AZA are similar to those observed in previous studies that have examined the role of DNA methylation in hippocampal-dependent learning paradigms [23], [30], it is worth noting that outside of the CNS 5-AZA is considered an S-phase specific compound that inhibits DNA methylation during DNA replication. Thus, the precise mechanism by which 5-AZA works in post-mitotic cells of the CNS is presently unknown. However, several studies have shown that 5-AZA can effectively modulate DNA methylation in the hippocampus [30] and prefrontal cortex [35]. Additional experiments will be required to determine how 5-AZA is affecting the methylation of genes in the LA following fear conditioning.

Given that DNA methylation is thought to negatively regulate transcription, the precise mechanism by which DNMT inhibition impairs memory consolidation remains paradoxical and poorly understood. Recent findings, however, have suggested that dynamic methylation of memory suppressor genes, such as PP1, may play a critical role. Contextual fear conditioning, for example, has been shown to lead to a dramatic increase in the methylation of the PP1 gene and a corresponding decrease in PP1 gene expression in the hippocampus during the consolidation period following training [30]. In the presence of DNMT inhibitors, however, methylation of the PP1 gene is significantly reduced, with a corresponding increase in PP1 gene expression [30]. Thus, it appears that one mechanism by which DNMT inhibitors impair synaptic plasticity and memory is by promoting the expression of memory suppressor genes that would otherwise be suppressed by training [23], [30], [34]. Interestingly, a second mechanism by which DNMT inhibition may regulate memory formation and synaptic plasticity is by influencing histone acetylation [23], [34]. In our own experiments, for example, we show that intra-LA infusion of 5-AZA not only impairs fear memory consolidation but also significantly attenuates the training-related increase in H3 acetylation following fear conditioning. Further, pre-treatment with the HDAC inhibitor TSA was observed to rescue the memory consolidation deficit induced by the DNMT inhibitor 5-AZA. A similar pattern of findings was observed in our neurophysiology experiments, where co-application of TSA to amygdala slices completely reversed the 5-AZA-infuced LTP deficit at both thalamic and cortical inputs to the LA. Future studies will be required to understand how histone acetylation and DNA methylation interact to regulate memory formation in the LA.

In summary, the findings of the present study clearly suggest that histone acetylation and DNA methylation are critical for fear memory consolidation and synaptic plasticity in the LA. Our findings represent the first comprehensive look at the role of epigenetic mechanisms in amygdala-dependent learning and memory and associated synaptic plasticity, and make an additional contribution towards understanding the cellular and molecular processes underlying emotional memory formation in the amygdala.

## Materials and Methods

### 

#### Subjects

Adult male Sprague-Dawley rats (Harlan) were housed individually in plastic cages and maintained on a 12∶12 hr light/dark cycle. Food and water were provided *ad libitum* throughout the experiments.

#### Drugs

For behavioral experiments the histone deacetylase inhibitor Trichostatin A (TSA; Sigma, Cat. No. T8552), the DNA methyltransferase inhibitor 5-Aza-2′-deoxycytidine (5-AZA; Sigma, Cat. No. A3656), and the MEK inhibitor U0126 (Promega, Cat. No. V1121) were dissolved in 100% DMSO to yield a stock concentration of 4 µg/µl, and then diluted 1∶1 in ACSF to a final concentration of 2 µg/µl prior to infusion into the brain. For slice electrophysiology experiments 5-AZA was dissolved in DMSO to a stock concentration of 30 mM and diluted in ACSF to 30 µM prior to bath application. TSA was dissolved in DMSO to a stock concentration of 2.5 mM and diluted in ACSF to 2.5 µM prior to bath application.

#### Surgical procedures

Under a mixture of Ketamine (100 mg/kg) and Xylazine (6.0 mg/kg) anesthesia, rats were implanted bilaterally with 26-gauge stainless steel guide cannulas (Plastics One) aimed at the LA [Bregma −3.2 AP, ±5.0 ML, −8.0 DV]. Guide cannulas were fixed to screws in the skull using a mixture of acrylic and dental cement, and a 31-gauge dummy cannula was inserted into each guide to prevent clogging. Rats were given Buprenex (0.2 mg/kg) as an analgesic and given at least five days to recover prior to experimental procedures. All procedures were conducted in accordance to the National Institutes of Health *Guide for the Care and Use of Experimental Animals* and were approved by the Yale University Institutional Animal Care and Use Committee (Protocol #2010-10801).

#### Fear conditioning and Western blotting experiments

To examine the time course of H3/H4 acetylation and DNMT 3A/3B expression following fear conditioning, rats were habituated to handling and to the conditioning chambers for four days prior to training. On the training day, rats were conditioned with 3 tone-shock pairings consisting of a 20 sec, 5 kHz, 75 dB tone that co-terminated with a 1 sec, 1 mA foot shock (ITI = 120 sec). Rats were then sacrificed using an overdose of chloral hydrate (600 mg/kg) and decapitated at either 30, 60, or 90 min following training. Naïve rats were sacrificed on the same day without training. To examine the associative regulation of histone acetylation and DNMT expression following fear conditioning, rats assigned to naïve (“Naïve”), tone alone (“Tone Alone”), or paired (“Paired”) groups were habituated to handling and to the conditioning chambers for four days prior to training. Rats assigned to the immediate shock (“Imm. Shock”) condition were handled for 4 days without exposure to the conditioning chamber to prevent context learning [36]. On the training day, “Paired” rats were given 3 tone-shock pairings as described above. “Tone Alone” rats received 3 presentations of the tone in the absence of shock, while “Imm. Shock” rats received 3 shocks immediately upon introduction to the conditioning chamber. “Naïve” rats were sacrificed on the same day without training. Brains were frozen and stored at -80°C until processed.

For Western blotting, punches were taken from the LA using a 1 mm punch tool (Fine Science Tools, Foster City, CA) from 400-µm-thick sections cut on a sliding freezing microtome. Punches were manually dounced in 100 µl of ice-cold hypotonic lysis buffer [10 mM Tris-HCl, pH 7.5, 1 mM EDTA, 2.5 mM sodium pyrophosphate, 1 mM phenylmethylsulfonyl fluoride, 1 mM β-glycerophosphate, 1% Igepal CA-630, 1% protease inhibitor cocktail (Sigma) and 1 mM sodium orthovanadate]. Sample buffer was immediately added to the homogenates, and the samples were boiled for 4 min. Homogenates were electrophoresed on 18% (H3/H4) or 10% (DNMT3A/B) gels and blotted to Immobilon-P (Millipore, Bedford, MA). Western blots were blocked in 5% milk in TTBS buffer (50 mM Tris-HCl, pH 7.5, 150 mM NaCl, and 0.05% Tween 20) then incubated with anti-acetyl-histone H3 (1∶3K; Millipore), anti-histone H3 (1∶5K; Millipore), anti-acetyl-histone H4 (1∶5K; Millipore), anti-histone H4 (1∶5K; Millipore), anti-DNMT3A (1∶100; Santa Cruz), or anti-DNMT3B (1∶500; Cell Signaling) antibody. Blots were then incubated with anti-rabbit conjugated to horseradish peroxidase (1∶10K; Cell Signaling) and developed using West Dura chemiluminescent substrate (Pierce). GAPDH (1∶5K; Abcam) was used as a loading control for all Western blotting experiments. Optical densities of the bands were analyzed using NIH Image software. For analysis of H3/H4, optical densities for acetyl- or total histone H3/H4 were normalized to GAPDH for each sample and expressed as a percentage of that in the Naïve control group.

#### Behavioral experiments

Cannulated rats were habituated to handling, the conditioning chamber, and dummy cannula removal for 2 days prior to training. On the training day, rats were conditioned with either 2 (TSA experiment) or 3 (5-AZA experiment) tone-shock pairings consisting of a 20 sec, 5 kHz, 75 dB tone that co-terminated with a 1 sec, 0.5 mA (TSA) or 1 mA (5-AZA) foot shock (ITI = 120 sec). The weaker training protocol was used for the TSA experiment to avoid a ceiling effect, which might obscure our ability to detect enhanced memory consolidation following infusion of the HDAC inhibitor. One hour following training, rats were given intra-LA infusion of either 50% DMSO in ACSF [vehicle; containing (in mM): 115 NaCl, 3.3 KCl, 1 MgSO4, 2 CaCl2, 25.5 NaHCO3, 1.2 NaH2PO4, and 10 glucose], TSA (1 µg/side in 0.5 µL; 0.125 µL/min) or 5-AZA (1 µg/side in 0.5 µL; 0.125 µL/min). Infusion cannulas remained in the guides for 1 min following infusion to allow for drug diffusion from the tip. Testing for STM and LTM occurred at 2 and 24 hrs following training, respectively, in separate groups of rats. For each test, rats were placed in a distinct environment that was dark and consisted of a flat black plastic floor that had been washed with a peppermint-scented soap and were exposed to 3 conditioned stimulus (CS) tones (STM test) or 9 tones (LTM test).

In a separate behavioral experiment, we examined the ability of the HDAC inhibitor TSA to rescue the consolidation deficit induced by the DNMT inhibitor 5-AZA. Rats were trained with 3 tone-shock pairings consisting of a 20 sec, 5 kHz, 75 dB tone that co-terminated with a 1 sec, 1.0 mA foot shock. Immediately after training, rats received infusions of either TSA (1 µg in 0.5 µl/side) or vehicle (0.5 µl/side), followed 1 hr later by either 5-AZA (1 µg in 0.5 µl/side) or vehicle (0.5 µl/side). Rats were then tested for LTM the next day.

Freezing behavior, defined as a lack of all movement with the exception of that required for respiration, was recorded and expressed as a percent of the total CS presentation time. Freezing was scored using automated activity monitors (Coulbourne Instruments Model # H10-24A) mounted on the top of each behavior chamber. Data were analyzed with repeated-measures ANOVA. Differences were considered significant if *p*<0.05.

#### Pharmacological and Western blotting experiments

To examine whether training-related elevations in histone H3 acetylation and DNMT3A expression are downstream of ERK/MAPK signaling in the LA, rats received intra-LA infusion of either 50% DMSO vehicle or the MEK inhibitor U0126 (1 µg/side in 0.5 µL; 0.125 µL/min) 30 min prior to auditory fear conditioning consisting of 3 tone-shock pairings [20 sec, 5 kHz, 75 dB tone; 1 sec, 1 mA footshock] and were sacrificed 90 min after training. This infusion to training interval for U0126 has previously been shown to reliably impair memory consolidation of auditory fear conditioning [5]. To examine the effects of HDAC and DNMT inhibition on training-related elevations in histone acetylation, cannulated rats were habituated to handling, the conditioning chamber, and dummy cannula removal for 2 days prior to training as described above. On the training day rats receiving either 5-AZA or TSA (and their respective vehicle groups) were conditioned and infused as described above. Rats infused with 5-AZA or TSA were sacrificed 30 min following drug infusion (90 min following conditioning). Following each experiment, brains were frozen and stored at −80°C until processed for H3/H4 acetylation using Western blotting as described above. For analysis of H3/H4, optical densities for acetyl- or total histone H3/H4 were normalized to GAPDH for each sample and expressed as a percentage of that in the Vehicle control group.

#### Slice electrophysiology experiments

Three to five week old male Sprague Dawley rats were deeply anesthetized with Ketamine (100 mg/kg) and rapidly decapitated. Brains were quickly extracted and placed in a dish filled with oxygenated ice-cold ACSF. 400 µm-thick coronal sections containing the LA were cut using a Vibratome and collected in a chamber containing 32°C oxygenated ACSF for 30 min. Prior to recordings, slices were allowed to return to room temperature for at least one hour. An upright microscope equipped with infrared differential interference contrast optics (IR-DIC) was used to perform field recordings under visual guidance. Glass electrodes were filled with ACSF and had resistances of 4–8 MΩ. Stimuli (150 µsec duration) were delivered through bipolar stainless steel electrodes.

For “thalamic” recording experiments, stimulation electrodes were placed in the ventral striatum, just medial to the LA. This stimulating protocol activates fibers that originate, at least in part, in the auditory thalamus [37]. For “cortical” recording experiments, stimulation electrodes were placed in the external capsule, just dorsal to the LA. This stimulating protocol activates fibers that originate, at least in part, in the auditory cortex [38]. The stimulation was kept at a minimum and adjusted for each slice to produce a reliable field-evoked response that was ∼50% of the maximal amplitude response. Baseline responses were monitored at 0.06 Hz. Following stabilization of baseline responses, one of two different LTP-induction protocols was used. For TSA alone experiments, we used a relatively weak LTP induction protocol consisting of a 100-Hz tetanus given three times at 1-min intervals at test intensity. For 5-AZA alone and 5-AZA+TSA experiments, we used a stronger protocol consisting of a 100-Hz tetanus given three times at 1-min intervals at 50% higher stimulation intensity. In our hands, this latter 100 Hz protocol produces a reliable LTP that lasts for at least 60 min, whereas the weaker 100 Hz protocol declines to baseline within 30 min [39]. Recordings were made at baseline test intensity for an additional 30 min after LTP. Picrotoxin (50 µM) was included in the bath in all experiments to block fast GABAergic transmission. Each slice was recorded from only once, and thus control and drug conditions were always from different slices. Typically, both vehicle and drug conditions were run from separate slices from the same animal on the same day. In control experiments, slices were perfused with ACSF/50 µM picrotoxin vehicle alone.

Data were acquired using Slice software (http://www.cns.nyu.edu/~sanes/slice_software/) written for Igor (NIH DC00540). Field potentials were extracted from Igor and analyzed using Spike 2 software. In all experiments, the amplitude of the negative-going field potential was measured, and LTP for each time point was expressed as a percentage of the pre-induction baseline. For analysis, measurements of evoked responses during the last 10 min of the recording session were compared with the last 5 min of the baseline period using Student's *t*-tests.
